# One-Year Consistency in Lifetime Frequency Estimates and Functions of Non-Suicidal Self-Injury in a Clinical Sample

**DOI:** 10.3389/fpsyt.2020.00538

**Published:** 2020-06-16

**Authors:** Daiva Daukantaitė, Reid Lantto, Sophie I. Liljedahl, Marjolein Helleman, Sofie Westling

**Affiliations:** ^1^Department of Psychology, Lund University, Lund, Sweden; ^2^Department of Clinical Sciences Lund, Psychiatry, Lund University, Clinical Psychiatric Research Center, Region Skåne, Lund, Sweden; ^3^Region Västra Götaland, Psykiatri Affektiva, Department of Psychiatry, Sahlgrenska University Hospital, Gothenburg, Sweden; ^4^Institute of Neuroscience and Physiology, University of Gothenburg, Sahlgrenska Academy, Gothenburg, Sweden; ^5^School of Nursing, Hanze University of Applied Sciences, Groningen, Netherlands

**Keywords:** self-injury, the Inventory of Statements About Self-Injury (ISAS), self-report measure, lifetime assessment, psychiatric, clinical

## Abstract

Non-suicidal self-injury (NSSI), the direct, deliberate destruction of one’s own bodily tissue in the absence of an intent to die, is frequently used for evaluating treatment in clinical care. One instrument for assessing NSSI is the Inventory of Statements About Self-Injury (ISAS). The ISAS is a self-rating measure examining the lifetime frequencies of NSSI behaviors and further exploring NSSI functions. The study aimed to examine the consistency of self-reported lifetime NSSI frequencies and functions (*via* the ISAS) in a clinical sample of individuals with current self-harm and/or recurrent suicidal behaviors over one year. Fifty-two individuals (84.6% women) completed the ISAS three times over 1 year. We found relatively good test-retest stability for most NSSI behaviors and functions, but the correlation coefficients and frequencies of NSSI behaviors varied substantially. Approximately, 50% of participants reported lower lifetime frequencies of NSSI behaviors at the later time points, with approximately 20% reporting a significant reduction in their lifetime frequencies over one year. This unexpected finding raises concerns about the accuracy of reporting lifetime NSSI frequencies among individuals with multiple psychiatric diagnoses and extensive NSSI behaviors across their lives. Further research is needed to determine more reliable ways of collecting data on the lifetime frequency of NSSI in clinical samples and the accuracy of lifetime NSSI frequency estimates in general.

## Introduction

Self-injurious behavior, suicidal or non-suicidal, is a common symptom of many different psychiatric disorders (1). Changes in frequency of self-injurious behavior are a common outcome measure in clinical studies and care (2–5). Deliberate self-harm (DSH) is often used as a broader term for self-injurious behavior, describing direct and indirect behaviors that jeopardize the self regardless of suicidal intent (6, 7). To evaluate treatment or obtain information useful for implementing treatment, a valid and reliable measure of the frequency and/or changes in the frequency of self-injurious behaviors over time is crucial. Over the last few decades, various measurement instruments have been developed to assess different types of DSH. Some instruments {e.g., Suicide Attempt Self-Injury Interview [SASII; (8)]; Self-Injurious Thoughts and Behaviors Interview [SITBI; (9)]} that assess multiple factors related to self-harm and suicide attempts are designed to be administered in a structured interview format, whereas others—which tend to be less comprehensive [e.g., the Self-Injury Questionnaire, (10)]—use a self-report format, require little time to administer, and are used in both clinical and non-clinical research. In this study, we will focus on non-suicidal self-injury (NSSI), a subcategory of DSH that represents the direct, deliberate destruction of one’s own bodily tissue (e.g., cutting, burning, carving) in the absence of an intent to die (11). Although the majority of individuals who engage in NSSI lack the intention to die, this behavior remains one of the strongest predictors of attempted and completed suicide (12–17) and is a risk factor for increased all-cause mortality (14). Moreover, NSSI has also been incorporated in the DSM-5, the American Psychiatric Association’s diagnostic system (18), as a “condition for further study” (19–21).

Several self-report instruments [e.g., the DSH Inventory (DSHI) (22)]; the Functional Assessment of Self-Mutilation [FASM; (23)]; the Inventory of Statements About Self-Injury [ISAS; (24)]) have been developed to assess NSSI. These instruments ask about specific forms of self-injurious behavior, such as cutting, carving, burning, biting, and punching oneself. While the original DSHI and the ISAS assess lifetime frequency of NSSI behaviors, the FASM requires individuals to report NSSI over the past year. The latter two instruments also evaluate the functions of the NSSI. Although the choice of instrument depends largely on the application of the knowledge of self-injurious behavior, a lifetime assessment of self-injurious behavior is generally of great interest because it enables assessment of the prevalence of NSSI. Furthermore, since lifetime NSSI remains an important risk factor for suicide even if an individual ceases NSSI (25), inaccurate reports of lifetime NSSI or assessing NSSI only in the last year might have decisive consequences for the individual. Inaccurate reports of lifetime NSSI frequencies could also be misleading when evaluating treatment for an individual seeking health care, which, in turn, could lead to inadequate or absent treatment or even incorrect evaluation of a new intervention. However, despite a surge in NSSI studies over the last decades, few examined the longitudinal consistency of lifetime NSSI frequency estimates and functions. Further, most of the research on NSSI has been completed on non-clinical samples. Existing self-report questionnaires might not be suitable for providing valid information on the lifetime nature and frequency of NSSI among individuals with multiple comorbidities, as well as extensive NSSI histories.

The ISAS, one of the most commonly used self-report instruments in self-harm research, comprises two parts: Part 1 assesses the lifetime frequency of 12 predefined NSSI behaviors (e.g., cutting, biting, and burning) and Part 2 assesses two main categories of NSSI functions (intrapersonal and interpersonal). Intrapersonal functions include motivations for NSSI that are independent of the individual’s surroundings, such as for regulating emotions, punishing themselves, or reducing suicidality. Conversely, interpersonal functions represent motivations stemming from the individual’s surroundings, such as for influencing others, establishing interpersonal boundaries, or bonding with others (24).

While the psychometric properties of the ISAS have been tested cross-sectionally in several countries (e.g., Sweden, Mexico, Spain, Korea, and Turkey) in both non-clinical (26–28) and clinical (29) samples, few studies have examined the consistency and stability of ISAS-measured lifetime frequency estimates and functions of NSSI over a period longer than several weeks or months. To the best of our knowledge, only Glenn and Klonsky (30) established the 1-year test-retest reliability of both parts of the ISAS in a sample of 51 undergraduate students with NSSI. Although the reported frequencies of NSSI behaviors varied substantially between the two measurement points in their study, Glenn and Klonsky (30) did not discuss the trustworthiness of these self-reported frequencies. Instead, they focused on the test-retest correlations between the two measurements of the 12 NSSI behaviors and functions, concluding that both parts of the ISAS demonstrate good stability over one year in a student sample. Expanding on these findings, Victor et al. examined the longitudinal changes in ISAS-measured NSSI functions, among other factors, in a large sample of patients being treated in a partial hospitalization and intensive outpatient treatment program specifically for self-injury and other self-destructive thoughts and behaviors. Victor et al. (31) reported significant but moderate correlations over time for both intrapersonal (*r* = .53, *p* <.001) and interpersonal (*r* = .46, *p* <.001) functions, significant decreases [with effects sizes varying from very low (Cohen’s d = 0.10) to low (Cohen’s d = 0.25)] for both functions, and no significant difference in the decrease between functions. The study further supported the relatively good stability of NSSI functions over time in the clinical sample, even though the correlations were lower than were those reported by Glenn and Klonsky (30).

Although the notion of assessing the lifetime NSSI frequency is attractive and important for many researchers and clinicians, the accuracy of such assessments has not been widely discussed. In a study, comparing interview accounts of NSSI behaviors with medical records for incarcerated individuals with a history of self-harm, less than 40% of the participants with self-harm in their medical records disclosed their lifetime self-harm when directly asked (32). To our knowledge, no empirical article has noted the accuracy in discussing the self-reported assessment of lifetime frequencies of NSSI behaviors. However, a number of articles have raised concerns over the reporting of the lifetime prevalence of mental disorders (33–36). While Copeland et al. (35), Moffitt et al. (33), and Takayanagi et al. (34) have found evidence for an underestimation of the lifetime prevalence of mental disorders, Olino et al. (36) found higher lifetime prevalence estimates for several mental disorders. These findings indicate that, in general, lifetime prevalence estimates based on retrospective self-reports are susceptible to recall bias and other memory distortions.

The aim of this paper is to assess the consistency in ISAS-measured lifetime NSSI frequencies and functions in individuals with current episodes of self-harm and/or recurrent suicidal behavior, who exhibit at least three diagnostic criteria for borderline personality, and who have regular contact with mental health services. Based on previous research [i.e., (30, 31)], we expect that ISAS-measured lifetime frequencies of NSSI behaviors and functions will have a relatively good stability over a year in the clinical sample.

## Method

### Participants

One hundred twenty-five participants with current episodes of self-harm and/or recurrent suicidal behavior, as well as at least three diagnostic criteria for borderline personality disorder (BPD), were recruited from four psychiatric inpatient clinics in Skåne, Sweden, for a project evaluating the effects of Brief Admission to the hospital by self-referral (37). All participants were undergoing treatment at a psychiatric outpatient clinic throughout the study. Of those, 52 individuals (84.6% women) with complete data for the ISAS behavioral section (Part 1) at three time points were included in the study and comprised our analytical sample. An attrition analyses—performed by comparing participants with complete data on the ISAS behavioral scales at all three time points to those with incomplete data—did not show any significant differences in their lifetime frequency of NSSI behaviors and functions at T1, except that the distress function was endorsed by the participants in the analytical sample as more relevant (*M* = 2.60, *SD* = 1.60) compared to participants with incomplete data (*M* = 1.92, *SD* = 1.63; *t*(97) = 2.10, *p* = .039, Cohen’s *d* = 0.42).

**Table 1** shows the descriptive statistics of the analytical sample. Participants had up to seven diagnoses (median 3) as assessed by the Mini International Neuropsychiatric Interview (38) and Structured Clinical Interview for DSM-IV Axis II [SCID-II; (39)]. Twenty-four (46.2%) participants reported non-psychiatric disorders, among which hypothyreosis (n = 6) and asthma (n = 4) were the most common. Nine participants reported being diagnosed with attention deficit hyperactivity disorder (ADHD) and four reported an autism diagnosis.

**Table 1:** Baseline sociodemographic and clinical characteristics of the participants (N = 52).

Variable	Mean (SD)/No. (%)
Age, mean (SD)	33.2 (9.5)
Female, No. (%)	44 (84.6)
Education, No. (%)	
Elementary school or less	14 (26.9)
High school degree	26 (50.0)
Bachelor’s/Master’s degree or higher	12 (23.1)
Living alone, No. (%)	25 (48.1)
Living with partner, No. (%)	21 (40.4)
Child (-ren) at home, No. (%)	16 (30.8)
Clinical Characteristics	
Depressive Disorder, No. (%)	24 (46,2)
Suicide ideation, last month No. (%)	50 (96.2)
Suicidal behavior, last year No. (%)	41 (78.8)
Anxiety Disorders, No. (%)	14 (26.9)
Bipolar and related disorders, No. (%)	15 (28.8)
Post-Traumatic Stress Disorder, No. (%)	25 (48.1)
Obsessive-compulsive disorder, No. (%)	10 (19.2)
Eating Disorders, No. (%)	8 (15.4)
Substance-Related Disorders, No. (%)	21 (40.4)
Psychotic Disorders, No. (%)	4 (7.7)
Personality Disorder (Borderline excluded), No. (%)	36 (69.2)

### Procedure

This study was carried out in accordance with the latest version of the Declaration of Helsinki, and was approved by the Regional Ethical Board at Lund University (Dnr 2014/570). Participants were recruited from psychiatric in-patient and out-patient units in a region serving 1.3 M inhabitants. After providing informed consent, the participants completed the ISAS, which was administered as a self-report form online. A research assistant or the PI (a psychiatrist) was present during data collection, to answer possible questions or give support. Some participants with more severe difficulties concentrating asked if the forms could be read to them aloud. When requested, this help was provided. Data was collected at baseline (T1) and after 6 (T2) and 12 (T3) months (40).

### Measures

The ISAS (24, 30) is a self-rating measure of NSSI behavior. This measure contains two parts. Part 1 assesses the frequencies of different forms of self-injurious behavior with the following statement: *“Please estimate the number of times in your life you have intentionally (i.e., on purpose) performed each type of non-suicidal self-harm (e.g., 0, 10, 100, 500).”* This statement is followed by a list of 12 different forms of self-injurious behavior as well as one labeled “other” (see **Table 2** for a complete list of the forms of behavior). The internal consistency values (Cronbach’s alpha) of the ISAS behavioral scale were .89 (T1), .88 (T2), and .73 (T3). Responders are also asked to report a number of descriptive and contextual aspects of their behavior. These include the age of onset, whether they experience physical pain from NSSI, whether they are alone or together with others when they injure themselves, the length of time that usually passes between the first impulse to self-injure and performance of the actual act, and whether the individual wants to stop.

**Table 2:** Descriptive statistics and Spearman correlations for the ISAS behaviors across three time points.

NSSI behaviors	T1			T2			T3			Spearman correlations between NSSI behaviors at		
	M (SD)	Median	Range	M (SD)	Median	Range	M (SD)	Median	Range	T1 and T2	T2 and T3	T1 and T3
Cut	477.5 (1445.6)	68.5	10,000	400.9 (1394.7)	100	10,000	411.3 (1411.5)	50	10,000	.76***	.83***	.78***
Bite	133.2 (451.3)	3	2,500	51.60 (156.5)	3	1,000	88.6 (315.1)	5	2,000	.77***	.59***	.60***
Burn	226.1 (1384.9)	1.5	10,000	235.9 (1389.4)	1	10,000	68.6 (210.8)	2	1,000	.83***	.76***	.84***
Carve	366.0 (1402.7)	42.5	10,000	413.8 (1406.6)	72.5	10,000	453.5 (1545.5)	35	10,000	.33*	.61***	.38**
Pinch	197.4 (495.0)	5	2,500	100.7 (267.0)	2.5	1,500	61.4 (172.8)	5	1,000	.82***	.60***	.65***
Pull hair	71.6 (212.0)	0	1,000	226.3 (1385.5)	0	10,000	181.1 (973.4)	0	7,000	.57*	.80***	.59***
Severe scratch	141.3 (325.6)	10	1,700	134.9 (415.2)	7.5	2,500	166.0 (706.6)	10	5,000	.65***	.55***	.56***
Bang/Hit	347.6 (1409.5)	20	10,000	92.2 (179.6)	20	1,000	157.5 (385.3)	20	2,000	.76***	.76*	.79**
Interfere with wounds	408.2 (1441.7)	20	10,000	386.6 (1406.9)	45	10,000	529.1 (1606.2)	50	10,000	.54**	.82***	.56***
Rub skin	68.8 (222.9)	0	1,000	30.4 (142.6)	0	1,000	40.7 (155.2)	0	1,000	.63**	.68***	.59**
Stick self with needles	37.8 (141.2)	0	1,000	27.5 (66.7)	0	300	35.4 (100.1)	0	500	.63***	.71**	.65***
Swallow chemicals	21.6 (71.5)	0.5	500	14.8 (42.3)	0	200	27.1 (98.9)	0	500	.40**	.57***	.52***
Other	263.7 (1394.3)	0	10000	42.6 (118.3)	0	500	243.3 (1008.4)	0	7,000	.32*	.29*	.31*
NSSI behaviors, total	2760.7 (9019.2)	786.5	64181	2158.0 (7142.2)	670	51,628	2463.5 (5479.6)	499.5	33,200	.63***	.82***	.65***

*p < .05, **p < .01, ***p < .001.

Part 2 of the ISAS contains 39 items evaluating 13 different functions of self-harm (i.e., each function is represented by three items; see **Table 3** for a complete list of ISAS functions). Respondents who endorse some form of NSSI are asked to rate the relevance of each item to their experience of self-injury on a three-point Likert scale (*not relevant* = 0, *somewhat relevant* = 1, *very relevant* = 2). Following Klonsky and Glenn (24), the ISAS functions were grouped into two factors representing intrapersonal (e.g., *affect regulation*) and interpersonal (e.g., *autonomy*) functions. These two superordinate function scores were created by averaging the relevant subscales score (which ranged from 0 to 6). The internal consistency values (Cronbach’s alpha) of the ISAS functions are presented in **Table 3**.

**Table 3:** Means (SDs), Pearson correlations, and Cronbach’s alpha values for the ISAS functional scales across three time points.

ISAS function scales	T1		T2		T3		Pearson correlations between function scales at		
	M (SD)	α	M (SD)	α	M (SD)	α	T1 and T2	T2 and T3	T1 and T3
**Intrapersonal functions total scale**	3.71 (1.04)	.78	3.54 (0.91)	.81	3.59 (0.93)	.74	.67***	.63***	.52***
Affect Regulation	5.06 (0.90)	.12	4.76 (1.45)	.76	4.98 (0.97)	.29	.47***	.57***	.34*
Anti-Dissociation	3.26 (1.95)	.75	3.36 (1.62)	.69	3.23 (1.58)	.63	.60***	.68***	.53***
Anti-Suicide	3.46 (1.99)	.83	3.10 (2.10)	.90	3.29 (1.91)	.85	.56***	.70***	.64***
Marking Distress	2.66 (1.60)	.55	2.52 (1.77)	.68	2.64 (1.70)	.67	.74***	.68***	.54***
Self-Punishment	3.96 (1.84)	.80	3.98 (1.78)	.82	3.71 (1.80)	.79	.66***	.59***	.57***
**Interpersonal functions total scale**	0.99 (0.65)	.79	0.91 (0.70)	.77	0.88 (0.72)	.82	.69***	.82***	.52***
Autonomy	0.77 (1.38)	.78	0.55 (1.11)^a^	.73	0.91 (1.57)^a^	.85	.59***	.79***	.46***
Interpersonal Boundaries	1.02 (1.44)	.80	0.89 (1.23)	.63	0.98 (1.32)	.62	.61***	.66***	.47***
Interpersonal Influence	1.37 (1.54)	.77	1.35 (1.62)	.81	1.48 (1.62)	.84	.52***	.81***	.62***
Peer Bonding	0.08 (0.34)	.66	0.18 (0.91)	.95	0.10 (0.31)	−.11	.09	.01	.32*
Revenge	0.46 (0.92)	.75	0.38 (1.06)	.84	0.42 (1.07)	.87	.37**	.57***	.16
Self-Care	2.80 (1.73)	.68	2.72 (1.97)	.78	2.65 (1.66)	.72	.70***	.67***	.70***
Sensation Seeking	0.73 (1.25)	.75	0.65 (1.09)	.50	0.47 (1.06)	.72	.64***	.47***	.38**
Toughness	0.85 (1.12)	.48	0.87 (1.24)	.79	0.81 (1.12)	.52	.24	.50***	.46***

^a^indicates a significant mean difference; *p < .05, **p < .01, ***p < .001.

### Statistical Analyses

All NSSI behavior variables were non-normally distributed (i.e., skewness was clearly above the commonly used cut-point range, between −1 and 1 (41); thus, Spearman correlations were used to calculate the relationships among ISAS behaviors at the three time points, while Friedman tests were performed to examine within-group differences in these ISAS behaviors over the time points. Pearson correlations were used to calculate the relationships between the ISAS functional scales and repeated ANOVAs were performed to study within-group differences on the ISAS functional scales across the three time points.

For the attrition and *post hoc* analyses, between-group comparisons were conducted using the Mann-Whitney *U* test and an independent-samples *t*-test for the ISAS behavioral and functional scales, respectively.

## Results

### NSSI Characteristics

Participants reported that their age of NSSI onset was between 4 and 45 years old (*M* = 14.6, *SD* = 7.8). Two participants did not report their age of NSSI onset. The time since their last episode of NSSI varied from 1 to 275 days (*M* = 41.7, *SD* = 53.3; *Mdn* = 25) at T1, 0 to 496 days (*M* = 81.0, *SD* = 108.1, *Mdn* = 47.5) at T2, and 1 to 549 days (*M* = 102.6, *SD* = 138.1, *Mdn* = 41.5) at T3. However, four, eight, and eight individuals at T1, T2, and T3, respectively, did not report or reported that they did not remember the time of their last episode. The vast majority of participants (77.8%, 78.8%, and 92.0% at T1, T2, and T3, respectively) reported engaging in NSSI when alone.

### Frequencies of NSSI Behaviors

As **Table 2** shows, individuals reported high lifetime frequencies of NSSI behaviors at all three time points. Although the mean frequencies varied widely for most NSSI behaviors, no significant differences between the time points were found for the whole sample.

When we examined the reported frequencies more closely, we found that a large number of participants reported lower frequencies over time. Specifically, 25 out of the 52 participants (48.1%) reported lower frequencies at T2 as compared to T1 (*M*_DIFF T1-T2_ = 1929.6, *SD* = 3530.7; *Mdn* = 450), 27 (51.9%) reported lower frequencies at T3 as compared to T2 (*M*_DIFF T2-T3_ = 1178.0, *SD* = 3499.9; *Mdn* =400), and 23 (44.2%) reported lower frequencies at T3 as compared to T1 (*M*_DIFF T1-T3_ = 2628.5, *SD* = 6460.8; *Mdn* = 900). Twelve of the 52 participants (21.2%) reported a statistically significant reduction in lifetime frequency of NSSI behaviors across the three time points (*M*_DIFF T1-T2_ = 2120.1, *SD* = 3809.5; *Mdn* =670; *M*_DIFF T2-T3_ = 1859.4, *SD* = 5246.5; *Mdn* = 92; and *M*_DIFF T1-T3_ = 3979.5, *SD* = 8707.1; *Mdn* = 1091.5; *χ^2^* = 18.0, *p* <.001).

**Table 2** also shows the Spearman correlations among the three time points for the 12 NSSI behaviors. The lowest correlations were found for carving (.33,.61, and.38 between T1 and T2, T2 and T3, and T1 and T3, respectively) and other (.32,.29, and.31 between T1 and T2, T2 and T3, and T1 and T3, respectively), while the highest correlations were found for burning (.83,.76, and.84 between T1 and T2, T2 and T3, and T1 and T3, respectively).

The participants generally reported engaging in several forms of NSSI; on average, they reported engaging in seven forms (*SD* = 3; range: 1–12) at all three time points. The most frequent forms were cutting (88.5% of the sample at all three time points) and carving (86.5% at all three time points). The least frequent forms were hair-pulling and rubbing one’s skin (about 35% of participants at one time point at least).

### Functions of NSSI

**Table 3** shows the descriptive statistics, internal consistency coefficients, and correlations across the three time points for the ISAS functional scale. Participants reported that the intrapersonal functions were more relevant for their NSSI at all three time points compared to the interpersonal functions. Of the intrapersonal functions, affect regulation and self-punishment were the two most commonly endorsed functions; however, affect regulation also showed the lowest test-retest stability among the intrapersonal functions.

Regarding the within-group comparisons, a significant mean difference was found only for the autonomy function. The results showed that individuals endorsed the function significantly less at T2 (*M* = 0.55, *SD* = 1.11) compared to T3 (*M* = 0.91, *SD* = 1.57), although the effect size was low (*Cohen’s d* = −0.27).

## Discussion

In this study, we aimed to assess the consistency of ISAS-measured lifetime NSSI frequencies and functions in individuals with recurrent self-harm and regular contact with mental health services. To the best of our knowledge, this is the first study to examine both the lifetime NSSI behaviors and functions as measured by the ISAS over a year in a clinical sample.

Regarding the NSSI behaviors, similar to Glenn and Klonsky (30), we found relatively good test-retest stability among the three time points for most NSSI behaviors, even though the correlation coefficients between the different time points for the separate NSSI behaviors varied substantially. The reported frequencies of NSSI behaviors also varied markedly, with about 50% of participants reporting lower frequencies at a later time point and about 20% reporting a significant reduction in their lifetime frequencies across one year. This finding was unexpected given that the actual lifetime frequency of self-harm can only increase over time. The finding therefore raises concerns about the trustworthiness of self-reported lifetime frequencies of NSSI behaviors in a clinical sample.

There are several possible explanations for this finding, with the clinical characteristics of the sample being the most obvious one. On average, our participants had four psychiatric diagnoses and had engaged in NSSI for approximately 20 years. For individuals with several concurrent psychiatric diagnoses and extensive and long-lasting engagement in self-harm, the lifetime prevalence estimates are potentially susceptible to recall bias and other memory distortions (42–46). Moreover, self-injurious behavior can also be cyclic rather than linear; in other words, it can be exhibited for periods of time, stopped, and then resumed (47), making it even more difficult to recall and calculate the lifetime frequency of such behaviors.

Furthermore, confronting individuals who suffer from moderate or severe self-harm with the impossible task of *counting the number of times* they have ever harmed themselves is bound to lead to frustration and other negative emotional responses, thereby potentially worsening their likelihood of recall and potentially causing further harm. A psychiatrist involved in data collection actually noticed that participants in our sample experienced frustration when reporting lifetime frequency of NSSI, feeling that it was an impossible task (i.e., counting something too numerous to count). This is a specific source of frustration for the target group of this study—individuals with extensive and long-lasting NSSI (often with childhood onset). Indeed, even individuals with less intensive self-harm may feel frustrated when confronted with the task of counting lifetime NSSI acts. The enormous range of frequencies of the NSSI behaviors reported at T2 by Glenn and Klonsky (30) may be indicative of such frustration. For instance, the increased range in the frequency of hair pulling from 300 (T1) to 100,000 (T2), may not be realistic. Further, since most individuals who engage in NSSI do so in private, which was also the case in the present study, and only a small proportion of individuals who engage in NSSI present to hospitals or other clinical services (48), it is impossible to validate the self-reported lifetime frequency of NSSI behavior against medical records or other records. One attempt to do so was made in a study on incarcerated adults, where the registration of self-injurious behaviors is more frequent; in that study, Borschmann et al. (32) found poor agreement between interview accounts of NSSI behaviors and medically verified self-harm. The authors suggested triangulating data from multiple sources to increase the accuracy of self-harm assessments.

It is also possible that the instructions on how to respond to the ISAS could be interpreted differently by different individuals over time. For example, when interpreting the word *frequency*, an individual might count each specific self-injury act as a unique contribution to lifetime frequency; alternatively, they might count only the number of sessions in a day in which one or multiple injuries occurred. These differing interpretations of self-report survey instructions might affect the accuracy of lifetime NSSI reports. Accordingly, the instructions for survey completion should be clarified prior to data collection.

Another possible explanation is an *initial elevation bias*, which was examined recently in four field studies by Shrout et al. (49). Shrout et al. (49) noticed that when making repeated measurements of self-reported symptoms in college students, the initial measurement seems biased toward higher ratings, after which the ratings decrease. Although the initial elevation bias was found in all four field studies, the generalizability of their findings is limited because all participants were students, the assessments were intensive (e.g., twice daily for 44 days), and the main research questions in all these studies pertained to the participants’ internal states and behaviors before and after an important exam. By contrast, our sample was clinical, the assessments were much less intensive, and all participants had previously reported self-harming behaviors to their clinicians and the principal investigator. Thus, the first measured assessment in our study was not the first report on self-harming behaviors from participants. Furthermore, as Shrout et al. (49) concluded, internal states, as reported in the field studies, might be more sensitive to the initial elevation bias as compared to behaviors, which were our target in the present study; thus, the initial elevation bias seems less likely in our study. Nevertheless, the initial elevation bias needs more attention in clinical samples.

In none of the studies evaluating ISAS-measured lifetime NSSI behaviors were the raw frequencies discussed extensively. In most cases, the frequencies were further grouped either into “numeric groups” (i.e., 0 times, 1–2 times, 3–10 times, and more than 10 times), as in a study by Klonsky and Olino (50), or into ill-defined categories (i.e., “none”, “few”, “moderate”, and “common”) as in the studies by Bildik et al. (26) and Kim et al. (28). These studies again raise the question of the purpose of asking about exact lifetime NSSI frequency estimates. Changing the response format to a set of predefined ranges, for example—as in the aforementioned study by Klonsky and Olino (50) or as suggested by other researchers [e.g., 0, 1, 2–5, 6–20, ≥ 21 NSSI acts; (16, 51) or 0, 1, 2–10, 11–50, ≥ 51 NSSI acts, (52)]—could make respondents’ task more realistic. In most cases though, the suggested response formats for non-clinical samples appear to be arbitrary (varying among the studies) and lacking a clear theoretical rationale, which further complicates the validity and generalizability of the results. Furthermore, while this would certainly increase the trustworthiness of the reports, it might fail to capture the lifetime frequencies or changes in frequencies during a specific treatment, as any changes in the frequencies of ≥10, ≥21, or ≥ 51 would be missed, thereby making the instrument less suitable for clinical samples with severe self-injurious behaviors. Moreover, for most clinical samples, including the present sample, this would not be discriminative because the majority of our participants (about 90% of the sample) would be assigned to the highest category (i.e., ≥51 NSSI acts at all three time points). The predefined ranges therefore must be adapted to clinical samples with self-harm. One possible frame would be to use ranges that provide a normal distribution in a representative sample and validate them in relation to levels of psychopathology. Taking all this together, there might be a need for a different theoretical model and accompanying self-report lifetime assessment of NSSI in clinical populations with high frequencies of NSSI.

In line with the findings of Glenn and Klonsky (30) and Victor et al. (31), NSSI functions also showed relatively good stability over a year, with affect regulation being the most often endorsed function at all three time points. This finding is consistent with robust evidence that affect regulation is the most frequently endorsed function of NSSI [for a review, see Klonsky (53)] in both clinical [e.g., (29)] and nonclinical samples [e.g., (26)]. However, the stability of this function measurement was the lowest in Glenn and Klonsky’s study (30). It was also the lowest in the present study, among the intrapersonal functions. The recency of NSSI was suggested by Glenn and Klonsky as a possible explanation for this result. However, neither Glenn and Klonsky nor our author group found a clear and significant relationship between affect regulation and the recency of NSSI. Still, it is important to note that in both studies the small sample size did not allow for more extensive investigation. In the present study, there was a large variation in time since participants’ last engagement in NSSI (1–275 days at T1, 0–496 days at T2, and 1–549 days at T3), and only five participants reported rather recent (during the last 7 days) engagement in NSSI at all three time points. Although a *post hoc* analysis revealed no significant differences in the endorsement of the affect regulation function between those with recent engagement in NSSI and those with more distal engagement, those with more recent engagement indicated stronger endorsement of affect regulation than did those with more distal engagement. Moreover, the effect sizes were large for differences at T2 (Cohen’s d = 0.76) and T3 (Cohen’s d = 0.74), but not at T1 (Cohen’s d = 0.28) between the two groups. Therefore, the importance of the recency of NSSI for the endorsement of affect regulation remains an open question that needs further consideration in larger clinical and non-clinical samples. Furthermore, while a lifetime period is given for assessing the frequency of NSSI behaviors, no time frame is stipulated for assessing the functions. That is, individuals with rather recent engagement in NSSI could think about the most recent NSSI incident when evaluating the relevance of certain functions, whereas those with more distal engagement might use a more general evaluation.

In this study, the affect regulation function showed very low internal consistency at both T1 and T3, even though the internal consistency values of the intrapersonal functions in general were acceptable or good at all three time points; this could also be a possible reason for the low test-retest correlations. None of the other reviewed studies, except Lindholm et al. (29), reported Cronbach’s alpha values for the 13 functions (they instead reported alpha values only for the total function scales). It is therefore not possible to determine if this is an unexpected result found only in the present study, or if it was the case in other studies. However, the test-retest correlations did not improve even when we re-calculated the correlations after dropping the problematic item and the alpha values for the remaining two items increased at all three time points.

### Limitations

First, our results might be confounded by the duration and severity of illness, which over time could influence the motives, cognition, and affect associated with NSSI. Second, the present study can only provide tentative conclusions, given its limited generalizability due to a small sample size and skewed gender representation. The rather small sample size led to less than desirable statistical power to detect some differences (e.g., examining the relationship between affect regulation and the recency of NSSI) and study some effects (e.g., the moderating effect of early onset on changes in lifetime NSSI frequency). Furthermore, although clinical samples of self-harming individuals are predominantly made up of women, research has indicated that NSSI is also a problem among men; however, it might manifest differently in men than in women [see, e.g., (54, 55)].

### Conclusions

The results of the current study suggest that the lifetime self-reporting of NSSI behaviors and functions for individuals with a history of extensive self-harm, and perhaps particularly for those with an early onset and who have been diagnosed with several psychiatric disorders, might be of limited accuracy. Taken together, our results imply a need to develop a theoretical framework and accompanying self-report assessment for NSSI with clinically valid numeric categories of NSSI in populations with high frequencies of NSSI. Doing so may help in reliably assessing the lifetime frequency of NSSI behaviors and functions in clinical populations with severe and repeated self-harm.

## Data Availability Statement

The datasets generated for this study are available on request to the corresponding author.

## Ethics Statement

The studies involving human participants were reviewed and approved by The Regional Ethical Board at Lund University (Dnr 2014/570). All participants provided their written informed consent to participate in this study.

## Author Contributions

DD, SL, MH, and SW designed the larger project within which this study was conducted and wrote the protocol for it. SW organized the data collection. DD, RL, and SW conducted literature searches, provided summaries of previous research studies, and formulated the research questions for the present study. DD conducted the statistical analyses. DD, RL, and SW wrote the first full draft of the manuscript. All authors worked on several edits of the paper. All authors contributed to the article and approved the submitted version.

## Funding

This study was supported by grants from the Mats Paulsson Foundation, the Swedish Research Council, the Swedish National Self-Injury Project, regional research funds (Södra Regionvårdsnämnden), the Söderström-Königska Foundation, the Ellen and Henrik Sjöbring Foundation, the OM Persson Foundation, and the Maggie Stephens Foundation.

## Conflict of Interest

The authors declare that the research was conducted in the absence of any commercial or financial relationships that could be construed as a potential conflict of interest.

## References

[1] Haw C Hawton K Houston K Townsend E Psychiatric and personality disorders in deliberate self-harm patients. Br J Psychiatry (2001) 178(1):48–54. 10.1192/bjp.178.1.48 11136210

[2] Linehan MM Armstrong HE Suarez A Allmon D Heard HL Cognitive-behavioral treatment of chronically parasuicidal borderline patients. Arch Gen Psychiatry (1991) 48(12):1060–4. 10.1001/archpsyc.1991.01810360024003 1845222

[3] Bateman A Fonagy P Effectiveness of partial hospitalization in the treatment of borderline personality disorder: a randomized controlled trial. Am J Psychiatry (1999) 156(10):1563–9. 10.1176/ajp.156.10.1563 10518167

[4] Gratz KL Gunderson JG Preliminary data on an acceptance-based emotion regulation group intervention for deliberate self-harm among women with borderline personality disorder. Behav Ther (2006) 37(1):25–35. 10.1016/j.beth.2005.03.002 16942958

[5] McMain SF Links PS Gnam WH Guimond T Cardish RJ Korman L A randomized trial of dialectical behavior therapy versus general psychiatric management for borderline personality disorder. Am J Psychiatry (2009) 166(12):1365–74. 10.1176/appi.ajp.2009.09010039 19755574

[6] Hawton K Bergen H Casey D Simkin S Palmer B Cooper J Self-harm in England: a tale of three cities. Multicentre study of self-harm. Soc Psychiatry Psychiatr Epidemiol (2007) 42(7):513–21. 10.1007/s00127-007-0199-7 17516016

[7] Muehlenkamp JJ Claes L Havertape L Plener PL International prevalence of adolescent non-suicidal self-injury and deliberate self-harm. Child Adolesc Psychiatry Ment Health (2012) 6:10. 10.1186/1753-2000-6-10 22462815 PMC3348041

[8] Linehan MM Comtois KA Brown MZ Heard HL Wagner A Suicide Attempt Self-Injury Interview (SASII): development, reliability, and validity of a scale to assess suicide attempts and intentional self-injury. Psychol Assess (2006) 18(3):303–12. 10.1037/1040-3590.18.3.303 16953733

[9] Nock MK Holmberg EB Photos VI Michel BD Self-Injurious Thoughts and Behaviors Interview: development, reliability, and validity in an adolescent sample. Psychol Assess (2007) 19(3):309–17. 10.1037/1040-3590.19.3.309 17845122

[10] Santa Mina EE Gallop R Links P Heslegrave R Pringle D Wekerle C The Self-Injury Questionnaire: evaluation of the psychometric properties in a clinical population. J Psychiatr Ment Health Nurs (2006) 13(2):221–7. 10.1111/j.1365-2850.2006.00944.x 16608478

[11] Nock MK Why do people hurt themselves? New insights into the nature and functions of self-injury. Curr Dir psychol Sci (2009) 18(2):78–83. 10.1111/j.1467-8721.2009.01613.x 20161092 PMC2744421

[12] Asarnow JR Porta G Spirito A Emslie G Clarke G Wagner KD Suicide attempts and nonsuicidal self-injury in the treatment of resistant depression in adolescents: findings from the TORDIA study. J Am Acad Child Adolesc Psychiatry (2011) 50(8):772–81. 10.1016/j.jaac.2011.04.003 PMC3143365 21784297

[13] Chan MK Bhatti H Meader N Stockton S Evans J O’Connor RC Predicting suicide following self-harm: systematic review of risk factors and risk scales. Br J Psychiatry (2016) 209(4):277–83. 10.1192/bjp.bp.115.170050 27340111

[14] Hawton K Bale L Casey D Shepherd A Simkin S Harriss L Monitoring deliberate self-harm presentations to general hospitals. Crisis (2006) 27(4):157–63. 10.1027/0227-5910.27.4.157 17219747

[15] Wilkinson P Kelvin R Roberts C Dubicka B Goodyer I Clinical and psychosocial predictors of suicide attempts and nonsuicidal self-injury in the Adolescent Depression Antidepressants and Psychotherapy Trial (ADAPT). Am J Psychiatry (2011) 168(5):495–501. 10.1176/appi.ajp.2010.10050718 21285141

[16] Whitlock J Muehlenkamp J Eckenrode J Purington A Baral Abrams G Barreira P Nonsuicidal self-injury as a gateway to suicide in young adults. J Adolesc Health (2013) 52(4):486–92. 10.1016/j.jadohealth.2012.09.010 23298982

[17] Chesin MS Galfavy H Sonmez CC Wong A Oquendo MA Mann JJ Nonsuicidal Self-Injury Is Predictive of Suicide Attempts Among Individuals with Mood Disorders. Suicide Life Threat Behav (2017) 47(5):567–79. 10.1111/sltb.12331 PMC5724372 28211201

[18] American Psychiatric Association Diagnostic and statistical manual of mental disorders. 5th ed. Washington, DC: Author; (2013).

[19] Plener PL Fegert JM Nonsuicidal self-injury: a condition for further study. Child Adolesc Psychiatry Ment Health (2015) 9(1):30. 10.1186/s13034-015-0067-2 27408620 PMC4940976

[20] Shaffer D Jacobson C Proposal to the DSMV childhood disorder and mood disorder work groups to include non-suicidal self-injury (NSSI) as a DSM-V disorder. Am Psychiatr Assoc (2009).

[21] Gargiulo A Plener PL Baus N Margherita G Brunner R Kaess M Non-suicidal self-injury (NSSI) and suicidal behavior disorder (SBD) in DSM-5. Minerva Psichiatr (2014) 55(2):83–90.

[22] Gratz KL Measurement of Deliberate Self-Harm: Preliminary Data on the Deliberate Self-Harm Inventory. J Psychopathol Behav Assess (2001) 23(4):253–63. 10.1037/t04163-000

[23] Lloyd-Richardson EE Perrine N Dierker L Kelley ML Characteristics and functions of non-suicidal self-injury in a community sample of adolescents. Psychol Med (2007) 37(8):1183–92. 10.1017/S003329170700027X PMC2538378 17349105

[24] Klonsky ED Glenn CR Assessing the functions of non-suicidal self-injury: Psychometric properties of the Inventory of Statements About Self-injury (ISAS). J Psychopathol Behav Assess (2009) 31(3):215–9. 10.1007/s10862-008-9107-z PMC5736316 29269992

[25] Hamza CA Stewart SL Willoughby T Examining the link between nonsuicidal self-injury and suicidal behavior: a review of the literature and an integrated model. Clin Psychol Rev (2012) 32(6):482–95. 10.1016/j.cpr.2012.05.003 22717336

[26] Bildik T Somer O Kabukcu Basay B Basay O Ozbaran B The validity and reliability of the Turkish version of the inventory of statements about self-injury. Turk Psikiyatri Derg (2013) 24(1):49–57. 10.5080/u6901 23446540

[27] Castro Silva E Benjet C Juárez García F Jurado Cárdenas S Lucio Gómez-Maqueo ME Valencia Cruz A Adaptación y propiedades psicométricas del Inventory of Statements About Self-injury en estudiantes mexicanos. Acta Investigación Psicológica (2016) 6(3):2544–51. 10.1016/j.aipprr.2016.08.004

[28] Kim S Kim Y Hur JW Nonsuicidal Self-Injury among Korean Young Adults: A Validation of the Korean Version of the Inventory of Statements about Self-Injury. Psychiatry Investig (2019) 16(4):270–8. 10.30773/pi.2019.01.23 PMC6504776 30947497

[29] Lindholm T Bjarehed J Lundh LG Functions of nonsuicidal self-injury among young women in residential care: a pilot study with the Swedish version of the Inventory of Statements about Self-Injury. Cognit Behav Ther (2011) 40(3):183–9. 10.1080/16506073.2011.565791 21877957

[30] Glenn CR Klonsky ED One-year test-retest reliability of the Inventory of Statements about Self-Injury (ISAS). Assessment (2011) 18(3):375–8. 10.1177/1073191111411669 21665881

[31] Victor SE Styer D Washburn JJ Functions of nonsuicidal self-injury (NSSI): Cross-sectional associations with NSSI duration and longitudinal changes over time and following treatment. Psychiatry Res (2016) 241:83–90. 10.1016/j.psychres.2016.04.083 27156029

[32] Borschmann R Young JT Moran P Spittal MJ Snow K Mok K Accuracy and predictive value of incarcerated adults’ accounts of their self-harm histories: findings froman Australian prospective data linkage study. CMAJ Open (2017) 5(3):E694–e701. 10.9778/cmajo.20170058 PMC5621944 28893844

[33] Moffitt TE Caspi A Taylor A Kokaua J Milne BJ Polanczyk G How common are common mental disorders? Evidence that lifetime prevalence rates are doubled by prospective versus retrospective ascertainment. Psychol Med (2010) 40(6):899–909. 10.1017/S0033291709991036 19719899 PMC3572710

[34] Takayanagi Y Spira AP Roth KB Gallo JJ Eaton WW Mojtabai R Accuracy of Reports of Lifetime Mental and Physical Disorders: Results From the Baltimore Epidemiological Catchment Area StudyAccuracy of Reports of Lifetime DisordersAccuracy of Reports of Lifetime Disorders. JAMA Psychiatry (2014) 71(3):273–80. 10.1001/jamapsychiatry.2013.3579 PMC4135054 24402003

[35] Copeland W Shanahan L Costello EJ Angold A Cumulative prevalence of psychiatric disorders by young adulthood: a prospective cohort analysis from the Great Smoky Mountains Study. J Am Acad Child Adolesc Psychiatry (2011) 50(3):252–61. 10.1016/j.jaac.2010.12.014 PMC3049293 21334565

[36] Olino TM Shankman SA Klein DN Seeley JR Pettit JW Farmer RF Lifetime rates of psychopathology in single versus multiple diagnostic assessments: comparison in a community sample of probands and siblings. J Psychiatr Res (2012) 46(9):1217–22. 10.1016/j.jpsychires.2012.05.017 PMC3411854 22739001

[37] Westling S Daukantaité D Liljedahl S Oh Y Westrin Å Flyckt L Effect of Brief Admission to Hospital by Self-referral for Individuals Who Self-harm and Are at Risk of Suicide. A Randomized Clinical Trial. JAMA Network Open (2019) 2(6):1–14. 10.1001/jamanetworkopen.2019.5463 PMC6563573 31173128

[38] Sheehan DV Lecrubier Y Sheehan KH Amorim P Janavs J Weiller E The Mini-International Neuropsychiatric Interview (M.I.N.I.): the development and validation of a structured diagnostic psychiatric interview for DSM-IV and ICD-10. J Clin Psychiatry (1998) 59(Suppl 20):22–33;quiz 4-57. 9881538

[39] Ryder AG Costa PT Bagby RM Evaluation of the SCID-II personality disorder traits for DSM-IV: coherence, discrimination, relations with general personality traits, and functional impairment. J Pers Disord (2007) 21(6):626–37. 10.1521/pedi.2007.21.6.626 18072864

[40] Liljedahl S Helleman M Daukantaitė D Westrin Å Westling S A standardized crisis management model for self-harming and suicidal individuals with three or more diagnostic criteria of borderline personality disorder: The Brief Admission Skåne randomized controlled trial protocol (BASRCT). BMC Psychiatry (2017) 17:220. 10.1186/s12888-017-1371-6 28619050 PMC5472925

[41] Hair JF Multivariate data analysis. Upper Saddle River, N.J.: Prentice Hall (1998).

[42] Maurex L Lekander M Nilsonne Å Andersson EE Åsberg M Öhman A Social problem solving, autobiographical memory, trauma, and depression in women with borderline personality disorder and a history of suicide attempts. Br J Clin Psychol (2010) 49(3):327–42. 10.1348/014466509X454831 19555523

[43] Jørgensen CR Berntsen D Bech M Kjølbye M Bennedsen BE Ramsgaard SB Identity-related autobiographical memories and cultural life scripts in patients with Borderline Personality Disorder. Conscious Cogn (2012) 21(2):788–98. 10.1016/j.concog.2012.01.010 22356875

[44] Hurlemann R Hawellek B Maier W Dolan RJ Enhanced emotion-induced amnesia in borderline personality disorder. Psychol Med (2007) 37(7):971–81. 10.1017/S0033291706009792 PMC2633116 17224096

[45] Beblo T Mensebach C Wingenfeld K Rullkoetter N Schlosser N Driessen M Subjective memory complaints and memory performance in patients with borderline personality disorder. BMC Psychiatry (2014) 14(1):255. 10.1186/s12888-014-0255-2 25214199 PMC4172827

[46] Teixeira S Machado S Paes F Velasques B Silva JG Sanfim AL Time perception distortion in neuropsychiatric and neurological disorders. CNS Neurol Disord Drug Targets (2013) 12(5):567–82. 10.2174/18715273113129990080 23844680

[47] Walsh BW Treating self-injury: A practical guide. 2nd ed. Vol. xvii New York, NY, US: The Guilford Press; (2012) p. 413–xvii.

[48] Rowe SL French RS Henderson C Ougrin D Slade M Moran P Help-seeking behaviour and adolescent self-harm: a systematic review. Aust N Z J Psychiatry (2014) 48(12):1083–95. 10.1177/0004867414555718 25335872

[49] Shrout PE Stadler G Lane SP McClure MJ Jackson GL Clavél FD Initial elevation bias in subjective reports. Proc Natl Acad Sci (2018) 115(1):E15. 10.1073/pnas.1712277115 29255039 PMC5776801

[50] Klonsky ED Olino TM Identifying clinically distinct subgroups of self-injurers among young adults: a latent class analysis. J Consult Clin Psychol (2008) 76(1):22–7. 10.1037/0022-006X.76.1.22 18229979

[51] Burke TA Hamilton JL Abramson LY Alloy LB Non-suicidal self-injury prospectively predicts interpersonal stressful life events and depressive symptoms among adolescent girls. Psychiatry Res (2015) 228(3):416–24. 10.1016/j.psychres.2015.06.021 PMC4540325 26165966

[52] Whitlock J Knox KL The Relationship Between Self-injurious Behavior and Suicide in a Young Adult Population. Arch Pediatr Adolesc Med (2007) 161(7):634–40. 10.1001/archpedi.161.7.634 17606825

[53] Klonsky ED The functions of deliberate self-injury: a review of the evidence. Clin Psychol Rev (2007) 27(2):226–39. 10.1016/j.cpr.2006.08.002 17014942

[54] Laye-Gindhu A Schonert-Reichl KA Nonsuicidal Self-Harm Among Community Adolescents: Understanding the “Whats” and “Whys” of Self-Harm. J Youth Adolesc (2005) 34(5):447–57. 10.1007/s10964-005-7262-z

[55] Inckle K Strong and Silent: Men, Masculinity, and Self-injury. Men Masculinities (2014) 17(1):3–21. 10.1177/1097184X13516960

